# Adsorption characteristics of Pb(II) using biochar derived from spent mushroom substrate

**DOI:** 10.1038/s41598-019-52554-2

**Published:** 2019-11-05

**Authors:** Qianlan Wu, Yang Xian, Zilin He, Qi Zhang, Jun Wu, Gang Yang, Xiaohong Zhang, Hui Qi, Jing Ma, Yinlong Xiao, Lulu Long

**Affiliations:** 1 College of Environmental science, Sichuan Agricultural University, Chengdu, 611130 China; 2 Sichuan Radiation Detection and Protection Institute of Nuclear industry (Sichuan Nuclear Emergency Technical Support Center), Chengdu, 610052 China; 3 College of Water Conservancy and Hydropower Engineering, Sichuan Agricultural University, Yaan, 625014 China

**Keywords:** Environmental chemistry, Pollution remediation

## Abstract

As a multifunctional material, biochar is considered a potential adsorbent for removing heavy metals from wastewater. Most biochars with high adsorption capacities have been modified, but this modification is uneconomical, and modifying biochar may cause secondary pollution. Thus, it is necessary to develop an efficient biochar without modification. In this study, spent *P. ostreatus* substrate and spent *shiitake* substrate were used as the raw materials to prepare biochar. Then, the physicochemical properties of the biochars and their removal efficiencies for Pb(II) were investigated. The results showed that the physicochemical properties (e.g., large BET surface area, small pore structure and abundant functional groups) contributed to the large adsorption capacity for Pb(II); the maximum adsorption capacities were 326 mg g^−1^ (spent *P. ostreatus* substrate-derived biochar) and 398 mg g^−1^ (spent *shiitake* substrate-derived biochar), which are 1.6–10 times larger than those of other modified biochars. The Pb(II) adsorption data could be well described by the pseudo-second-order kinetic model and the Langmuir model. This study provides a new method to comprehensively utilize spent mushroom substrates for the sustainable development of the edible mushroom industry.

## Introduction

In recent years, heavy metals have been introduced into the environment as a result of industrial production, energy utilization, mining, and the application of pesticides and fertilizers^1–3^. Lead (Pb), one of the most common heavy metals, is highly toxic, has a wide range of sources, and is nonbiodegradable; thus, Pb-containing wastewater has become a serious environmental problem^4^. Currently, the treatments of heavy metals mainly include chemical precipitation, ionic exchange, reverse osmosis and adsorption. Among these methods, adsorption is considered the most promising due to its simple process and high efficiency^5^. However, the widespread use of adsorption has been limited by the lack of environmentally friendly and economical adsorbents^6^.

Biochar is a fine-grained and porous substance produced by slow pyrolysis under a limited supply of O_2_ at relatively low temperatures (<900 °C), and it is often derived from agricultural and forestry waste, poultry manure, sawdust and algae^7,8^. Biochar is a potential adsorbent for pollutants due to its large surface area, porous structure, abundant surface functional groups and high cation exchange capacity (CEC)^9^. Compared with activated biochar and carbon, biochar is considered to be less expensive because it does not require an additional activation process^10–12^. An increasing number of studies have modified biochar to enhance its adsorption capacity. However, modification of biochar may cause waste and pollution from the chemical reagents, and modifying biochar increases the relevant costs^11^. Thus, it is necessary to find an unmodified biochar with a high adsorption capacity toward heavy metals. Previous studies have demonstrated that the raw materials substantially affect the physicochemical properties and heavy metal adsorption capacity of biochar; therefore, choosing an appropriate feedstock is critical for the development of an efficient biochar^13,14^.

China is one of the largest mushroom producers in the world, accounting for over 80% of the total global output per year^15^. The improper disposal and utilization of spent mushroom substrate (SMS) causes adverse environmental problems. According to previous studies, SMSs consist of numerous organic substances. When SMSs are pyrolyzed into biochar, the adsorbent has a large surface area and numerous microporous structures due to the decomposition of these components. Therefore, SMSs can be ideal raw materials for the preparation of biochar^16^. Previous studies have explored the utilization of SMSs. Sewu *et al*. used SMS biochar (SMSBC), biochar from 10% seaweed kelp added to SMS (SK10BC), and biochar from kelp seaweed extract added to SMS (ESBC) to remove a cationic dye. Both SK10BC and ESBC exhibited excellent adsorptive performance. The maximum adsorption capacities were 610.1 mg g^−1^ and 410.5 mg g^−1^, respectively; however, the additional treatment of the biochar increased the cost^17^.

In this paper, biochars prepared from spent *P. ostreatus* substrate and spent *shiitake* substrate were employed for wastewater treatment. The specific objectives were to (1) prepare and characterize the studied biochars, (2) assess their adsorption capacity for Pb(II) in wastewater, and (3) explore the internal adsorption mechanism of biochar. This paper aims to provide technical support for the utilization of SMSs and the removal of Pb(II).

## Materials and Methods

### Materials

The SMSs were obtained from an edible mushroom factory in Chengdu, China. The spent *P. ostreatus* substrate mainly consisted of bran (60%), corn cob (20%) and rice straw (20%). The cellulose, hemicellulose and lignin contents were 25.78%, 7.55% and 32.9%, respectively. The spent *shiitake* substrate mainly consisted of sawdust (80%) and bran (20%), and the cellulose, hemicellulose and lignin contents were 24.99%, 4.81% and 23.16%, respectively.

The SMSs were comminuted and screened through a 40-mesh sieve after being air dried, placed into crucibles with lids and then carbonized in a muffle furnace for 2 h at temperatures of 300 °C, 500 °C and 700 °C (in an oxygen-limited atmosphere). The spent *P. ostreatus* substrate-derived biochars were labelled PC300, PC500 and PC700; the spent *shiitake* substrate-derived biochars were labelled SC300, SC500 and SC700.

### Characterization

Yields of the biochars were calculated based on mass balance. The pH values of the supernatants of aqueous biochar solutions were measured, and the ratio of solid to water was 1:20. The elemental C, H, N, and O contents were measured using an elemental analyzer (MicroCube, Elementar, Germany). The porosity properties of the biochars were estimated based on the adsorption-desorption isotherms of N_2_ at 77 K. The specific surface area was determined using the Brunauer–Emmett–Teller (BET) method with an ASAP 2020 surface area pore analyzer. The t-plot method is used to calculate the pore volume. The Barret–Joyner–Halender (BJH) method was applied to calculate the volume and width of the micropores. In addition, the total pore volume is calculated by the volume of adsorbed liquid at a relative pressure of 0.99. The biochar samples were dispersed on the surface of a copper containing a conductive adhesive. Gold was sprayed onto the surfaces of samples, and the samples were analyzed by scanning electron microscopy (SEM). The samples were prepared by the KBr pressed-disk technique, and the Fourier transform infrared spectroscopy (FTIR) spectra of biochars were obtained using TENSOR27 (Bruker, Ettlingen, Germany) from 400–4000 cm^−1^ with a resolution of 8 cm^−1^. X-ray diffraction (XRD) analysis was conducted with an X-ray diffractometer (D/max 2500, Rigaku, Japan) at 40 kV and 40 mA (Cu Kα radiation).

### Adsorption experiments

The Pb(II) stock solution (1000 mg L^−1^) was prepared by dissolving 1.599 g of lead nitrate (Pb(NO_3_)_2_) in deionized water (1000 mL). The background solution was NaNO_3_ (0.01 mol L^−1^). The stock solution was diluted to 200 mg L^−1^ before the adsorption experiments. All adsorption experiments were carried out in 100-mL glass cone flasks with 50 mL of the Pb(II) stock solution. For each measurement, three parallel experiments and a blank experiment were performed.

To study the effect of biochar dose, different amounts of biochar (0.01, 0.02, 0.05, 0.1, 0.2 and 0.5 g) were added to 50 mL of the 200 mg L^−1^ Pb(II) stock solution. The effect of the initial pH was investigated by varying the solution pH from 2 to 6 after adding 0.05 g of biochar into 50 mL of the 200 mg L^−1^ Pb(II) stock solution. The flasks were maintained with constant shaking (at 160 rpm) at room temperature (25 ± 0.5 °C) in a vapor-bathing constant temperature vibrator for 12 h.

For adsorption kinetics studies, 0.05 g of biochar was weighed into 100-mL glass cone flasks and mixed with 50 mL of the 200 mg L^−1^ Pb(II) stock solution; then, the mixture was shaken for 30 min, 1, 2, 4, 8, 12 and 24 h (160 rpm, 25 ± 0.5 °C). Adsorption isotherms were obtained by adding 0.05 g of biochar to 50 mL of a Pb(II) solution at different concentrations (50, 100, 150, 200, 300 and 400 mg L^−1^) in 100-mL flasks. The flasks were maintained with constant shaking (at 160 rpm) at room temperature (25 ± 0.5 °C) in a vapor-bathing constant temperature vibrator for 12 h.

The concentration of the filtrate was determined by flame atomic absorption spectrometry (AAS, Thermo Solaar M6, Thermo Fisher Scientific Ltd., USA) after filtration and dilution, and the adsorption capacity and removal rate were calculated.

### Data analysis

Microsoft Office Excel (2010) and SPSS 20 was used for data analysis. The experimental data were fit in Origin 9.0.

The adsorption capacity (*Q*_*e*_) and the Pb(II) removal efficiency (*E*) are expressed as follows:

1 $${Q}_{e}=({C}_{0}-{C}_{e})\times V/M$$

2 $$E( \% )=({C}_{0}-{C}_{e})/{C}_{0}\times 100$$

where *C*_0_ and *C*_*e*_ (mg L^−1^) are the initial and equilibrium concentrations of Pb(II), respectively; *M* (g) is the amount of biochar; and *V* (mL) is the volume of the Pb(II) solution.

The kinetics results were analyzed by pseudo-first-order and pseudo-second-order kinetics models, which can be expressed as follows^18^:

3 $$\mathrm{ln}({Q}_{e}-{Q}_{t})=\,\mathrm{ln}\,{Q}_{e}-{k}_{1}t$$

4 $$t/{Q}_{t}=1/{k}_{2}{Q}_{e}^{2}+t/{Q}_{e}$$

where *Q*_*t*_ (mg g^−1^) is the adsorption capacity at time t (min), and *k*_1_ (min^−1^) and *k*_2_ (g mg^−1^ min^−1^) are the rate constants for pseudo-first-order and pseudo-second-order adsorption, respectively.

The adsorption isotherm results were analyzed by the Langmuir, Freundlich and Temkin models, which can be expressed as follows^19^:

5 $${C}_{e}/{Q}_{e}=1/({K}_{L}\ast {Q}_{m})+{C}_{e}/{Q}_{m}$$

6 $$\mathrm{ln}\,{Q}_{e}=\,\mathrm{ln}\,{K}_{f}+1/n\ast \,\mathrm{ln}\,{C}_{e}$$

7 $${Q}_{e}=B\,\mathrm{ln}\,A+B\,\mathrm{ln}\,{C}_{e}$$

where *Q*_*m*_ (mg g^−1^) is the maximum adsorption capacity of the biochar, *Q*_*e*_ (mg g^−1^) is the equilibrium adsorption capacity, *K*_*L*_(L mg^−1^) is the Langmuir constant, and *K*_*f*_ (mg g^−1^)(mg L^−1^)^1/n^ and 1/*n* are the Freundlich constants. B = RT/b_T_, b_T_ is the Temkin constant (J mol^−1^), A is the Temkin isotherm equilibrium binding constant (L g^−1^), R is the gas constant (8.3145 J mol^−1^ K^−1^), and T is the absolute temperature at 298 K.

## Results and Discussion

### Characterization of different biochars

As shown in Table 1, as the pyrolysis temperature increased from 300 to 700 °C, the yields of biochar ranged from 50.41 to 34.90% (PC) and from 43.45 to 33% (SC), the ash contents increased from 33.27 to 55.71% (PC) and from 28.02 to 42.28% (SC), and the pH values increased from 9.58 to 12.31 (PC) and from 10.22 to 12.07 (SC). The decrease in the yield at high pyrolysis temperatures could be ascribed to the loss of volatile components, and the high ash content of the biochar indicates the accumulation of inorganic minerals, which could raise the pH and CEC^20,21^. Previous studies have shown that a high pH value for biochar may result from the release of alkali salts and the loss of acidic functional groups^22^. The elemental composition of PC and SC is shown in Table 1; very small amounts (≤0.20%) of other elements (e.g., S, Ca, Mn, Ag, Cr, and Cd) were detected, and the contents of these elements are not given in the table. In terms of elemental composition, the C, H and O contents of PC and SC decreased with increasing pyrolysis temperature, and the loss of these components resulted from the dehydration and decarboxylation of cellulose, hemicellulose and lignin. The H/C molar ratio represents the aromaticity of the biochar, and the O/C and (O + N)/C molar ratios are related to the hydrophilicity and polarity of the biochar, respectively. The low H/C, O/C and (O + N)/C ratios of PC700 and SC700 indicated that biochars obtained at high pyrolysis temperatures had higher aromaticity and lower hydrophilicity and polarity than the other samples^23^.

**Table 1:** Elemental composition and molar ratios of the biochars.

Sample	Yield (%)	Ash (%)	Elemental analysis^a^				Molar ratio			pH
			C (%)	H (%)	N (%)	O (%)	H/C	O/C	(O + N)/C	
PC300	50.41	33.27	59.94	2.91	1.75	2.13	0.92	0.48	0.52	9.58
PC500	39.82	52.35	38.73	1.09	0.44	1.69	0.34	0.14	0.15	10.37
PC700	34.90	55.71	37.94	0.99	0.67	0.99	0.30	0.06	0.07	12.31
SC300	43.45	28.02	46.78	2.84	2.71	19.65	0.73	0.32	0.37	10.22
SC500	37.14	35.67	45.92	1.94	2.11	12.36	0.51	0.24	0.27	10.64
SC700	33.00	42.28	44.85	1.23	1.09	9.55	0.33	0.18	0.20	12.07

^**a**^The low-content elements were not shown in table.

**Figure 1:**
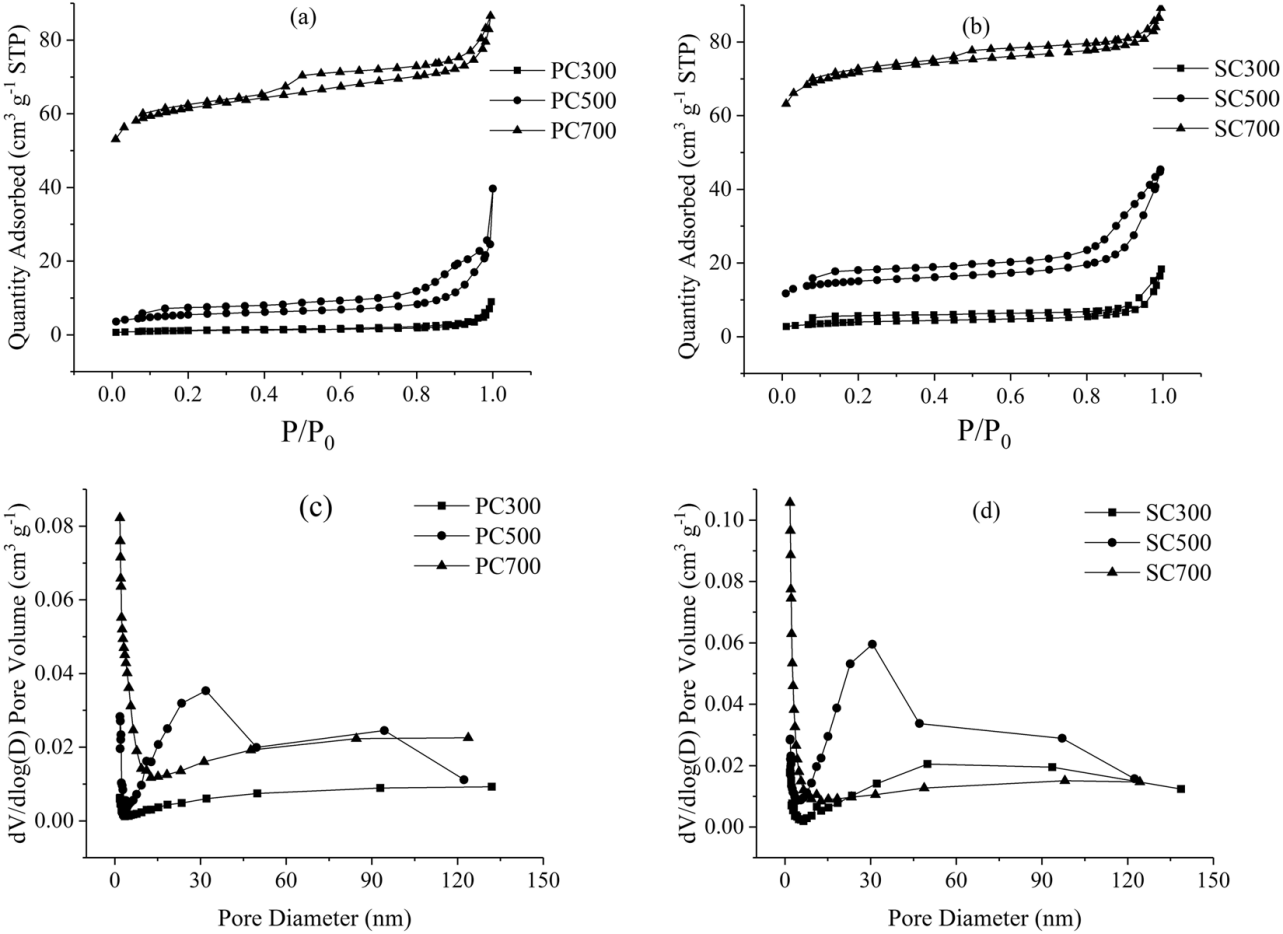
N2 adsorption-desorption isotherms and pore size distribution of PC and SC.

The N_2_ adsorption-desorption isotherms of biochar samples are shown in Fig. 1a,b. According to IUPAC classification, the nitrogen adsorption-desorption isotherms of PC and SC can be categorized as a mixture of type I and type IV. The hysteresis loop of the biochar isotherm at a relative pressure (P/P_0_) between 0.1 and 0.9 shows the existence of the microporous and mesoporous structures of PC and SC^12,24^. In Fig. 1c,d, the pore size calculated from the BJH method demonstrated that PC and SC have some wide pores and inhomogeneous textures. With the increase in pyrolysis temperature, the amount of mesopore and macropore volume decreased while the amout of micropore volume increased, this transformation from mesopore and macropore into micropore increased the surface area^25,26^. In this study, the BET surface area, micropore volume and total pore volume increased with increasing pyrolysis temperature, and the average pore size decreased (Table 2). The BET surface area of the biochars varied over a wide range from 3.79 to 188.57 m^2^ g^−1^ (PC) and from 12.97 to 218.70 m^2^ g^−1^ (SC). In particular, the surface areas of PC700 and SC700 are higher than those of previously reported modified biochars^25,27–30^. The SMSs in this study have high amounts of cellulose, hemicellulose and lignin after the cultivation of mushrooms, and these components contribute to the formation of the microporous structures and the inhomogeneous surfaces of the biochars. Previous studies stated that the increasing pyrolysis temperature took an active role in the adsorption of heavy metals. The organic matter of the biomass was completely carbonized at higher temperature; the surface area was greatly increased, providing more active sites for metal adsorption^31^. These results indicated that SMS-derived biochar may have good adsorption ability^32,33^_._

**Table 2:** Surface structural characteristics of the biochars.

Biochar	BET Surface Area (m^2^ g^−1^)	Micropore volume (cm^3^ g^−1^)	Total pore volume (cm^3^ g^−1^)	Average pore size (nm)
PC300	3.79	0.0002	0.014	14.66
PC500	18.05	0.0021	0.061	13.60
PC700	188.57	0.0750	0.134	2.84
SC300	12.97	0.0018	0.028	8.77
SC500	47.07	0.0153	0.070	5.97
SC700	218.70	0.0898	0.138	2.52

**Figure 2:**
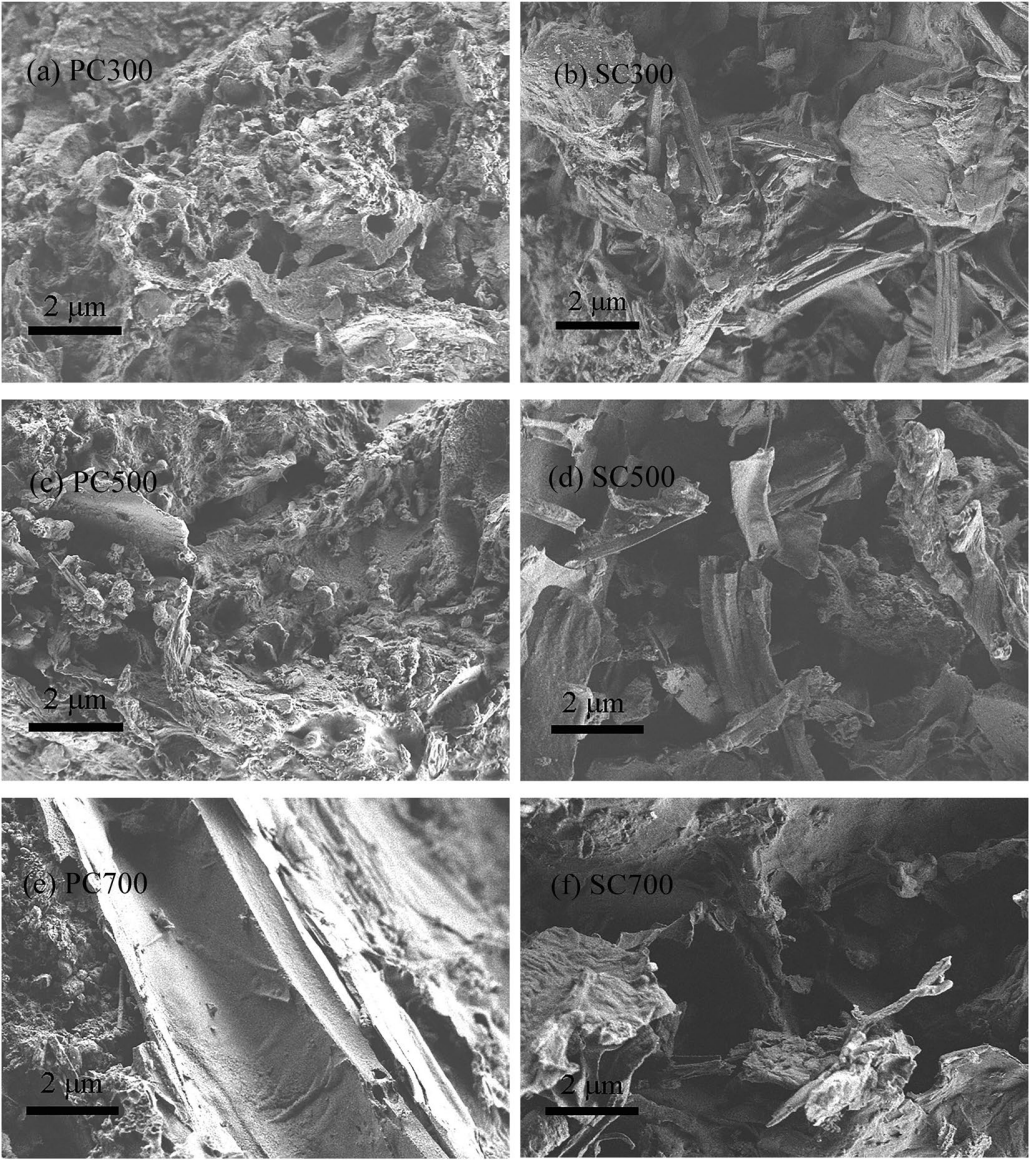
SEM images of the PC and SC samples.

SEM images of the biochars (Fig. 2) revealed different structures for the PC and SC samples. PC300 has several irregular deposits on its surface, but SC300 has several porous structures; PC500 has several tubular structures, but SC500 has a rough surface with more small porous structures; PC700 has a rough surface, and there are some stomata and dispersed small particles on the surface, but SC700 has several smooth areas among its tubular structures. The hollow rod-like structures and vertical channels of SC700 contributed to its BET surface area being larger than that of PC700, and thus, we can infer that SC700 may have a higher adsorption capacity than PC700. More microporous structures and a large number of particles formed at high pyrolysis temperatures, possibly due to the decomposition of cellulose, hemicellulose and other components. These results agree with the above BET analysis^26^.

### Pb(II) adsorption onto biochars

#### Effect of the adsorbent dose

**Figure 3:**
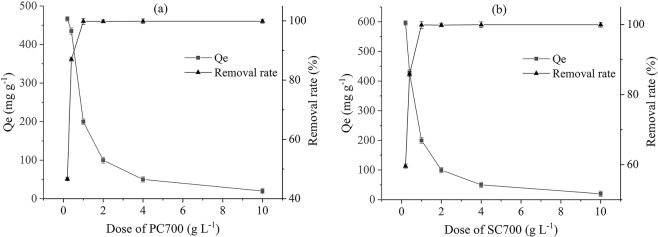
The effect of the adsorbent dose on Pb(II) removal (Represented by PC700 and SC700).

As the biochars displayed similar metal removal efficiency, the figures give only the trends of PC700 and SC700. As shown in Fig. 3, the biochars displayed excellent adsorption capacities at low dose, and the adsorption capacities of the PC and SC samples decreased with the increasing dose of adsorbent concentration. This result agrees with those reported by Zhou^4^. According to speculation, an increase in the adsorbent dose for a constant Pb(II) concentration limits the degrees of freedom of Pb(II), and the volume of the solution leads to saturation and the aggregation of active sites, which may reduce the adsorption capacity^34^.

#### Effect of the initial pH

**Figure 4:**
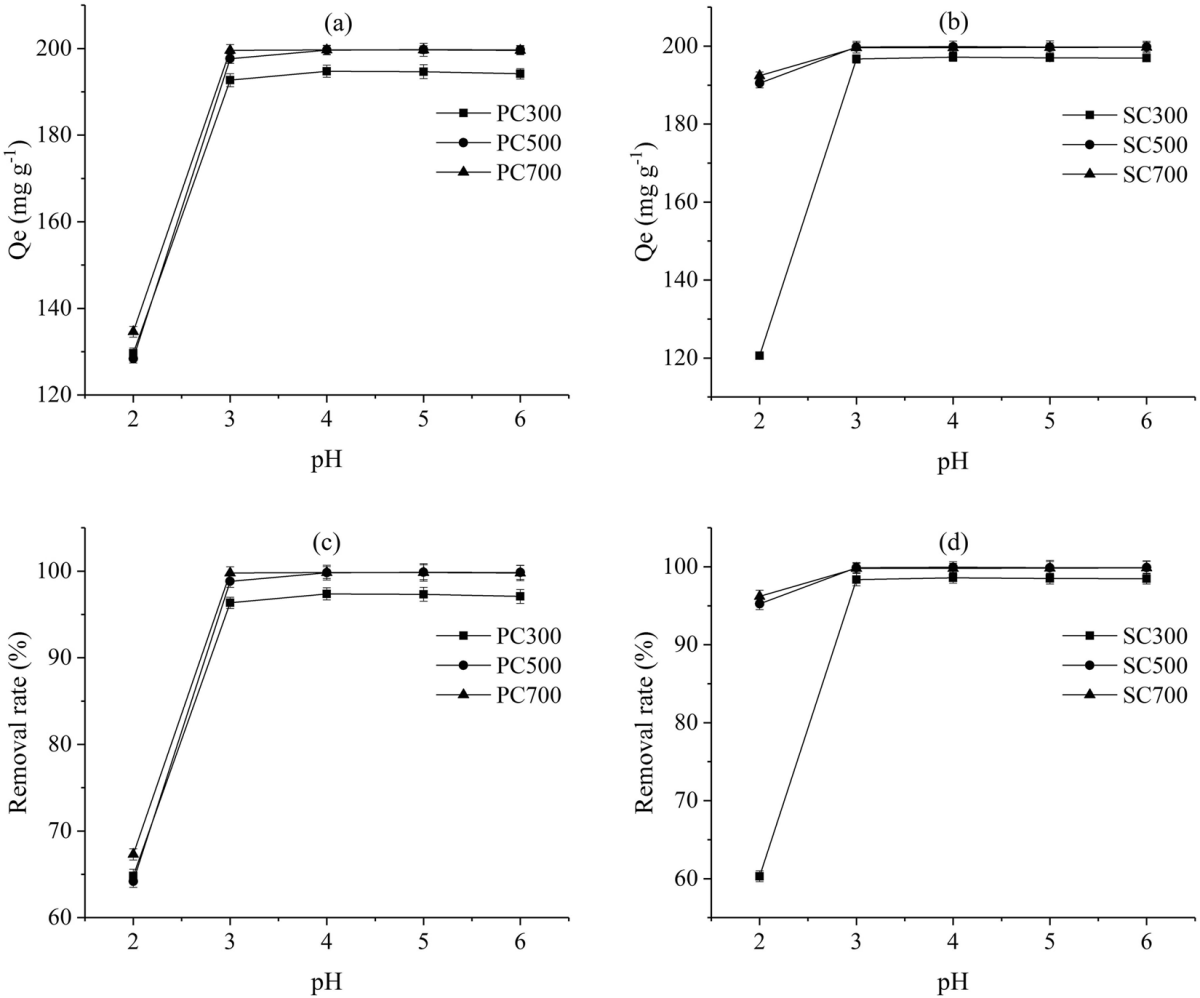
Effect of pH on the adsorption capacities of Pb(II) for the different biochars.

pH value is one of the important parameters in the adsorption process, it affects the solubility of metal ions in solution and the surface charges of biochar. To establish the influence of pH for the adsorption process, pH was varied between 2 and 6. With increasing pH, the adsorption capacity of Pb(II) for the PC and SC samples increased (Fig. 4). In particular, the adsorption capacity increased rapidly as the pH increased from 2 to 3, and the adsorption capacity was stable as the initial pH exceeded 3. A similar trend was reported by Luo^35^. The low adsorption capacities at low pH values resulted from the charge repulsion between the acidic functional groups on the biochars and Pb(II). As the pH gradually increased, the surface of the biochars became negatively charged due to the decrease in hydronium ions, making the biochar surfaces more available for Pb(II) and thus increasing the adsorption capacity^36^.

#### Kinetics

**Figure 5:**
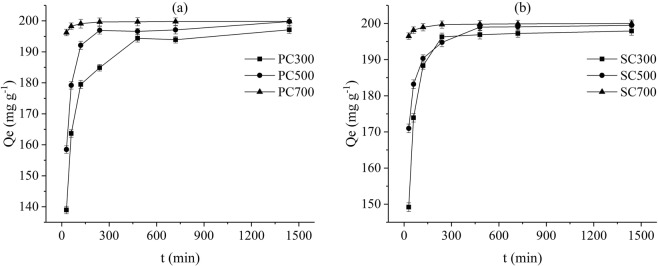
Adsorption kinetics of Pb(II) for the different SMS-derived biochars.

The pseudo-first-order model is based on the assumption that the adsorption rate is controlled by the diffusion of the adsorbate. The pseudo-second-order model relies on the assumption that the rate-limiting step is the chemical adsorption step due to surface adsorption interactions^37,38^. Thus, the pseudo-first-order and pseudo-second-order models were used to evaluate the adsorption of Pb(II) in this study. The adsorption of Pb(II) onto the biochars showed two distinct phases: a rapid phase over the first three hours and a second slow phase until reaching equilibrium at approximately 8 h (Fig. 5). According to the experimental data presented in Table 3, the determination coefficients (R^2^) of the pseudo-second-order model (0.984–0.996) are higher than those of the pseudo-first-order model (0.699–0.917), and the adsorption capacity (Qexp) values acquired from the pseudo-second-order model are closer to the experimental values (Qe). The well-fit data indicated that the pseudo-second-order kinetic model can be considered for Pb(II) adsorption onto the PC and SC samples, and the adsorption of Pb(II) may be due to chemisorption, adsorption processes involving chemical bonding between heavy metal ions and functional groups, which is also consistent with the results of a previous study^39^.

**Table 3:** Pseudo-first-order and pseudo-second-order model parameters.

Sample	Qexp (mg g^−1^)	Pseudo-first-order			Pseudo-second orde		
		Qe (mg g^−1^)	k_1_ (min^−1^)	R^2^	Qe (mg g^−1^)	k_2_ (g mg^−1^ min^−1^)	R^2^
PC300	197.14	189.54	0.0402	0.860	198.02	0.00040	0.996
PC500	199.79	195.60	0.0525	0.903	202.00	0.00062	0.984
PC700	199.81	199.40	0.1377	0.804	200.00	0.00893	0.993
SC300	197.91	194.68	0.0453	0.917	198.22	0.00048	0.990
SC500	199.48	195.29	0.0663	0.741	200.00	0.00096	0.993
SC700	199.98	199.43	0.1401	0.699	200.00	0.00909	0.995

#### Adsorption isotherm studies

The Langmuir model is an ideal monolayer adsorption model that assumes that all the adsorption sites have equivalent adsorption energies and that there are no mutual interactions between adsorbed molecules. The Freundlich model is suitable for adsorption onto heterogeneous surfaces, and the absorption capacity will increase continuously at high concentrations^40^. The Temkin model assumes that the heat of adsorption decreases linearly with the adsorption coverage due to the interaction of adsorbent and adsorbate.

**Figure 6:**
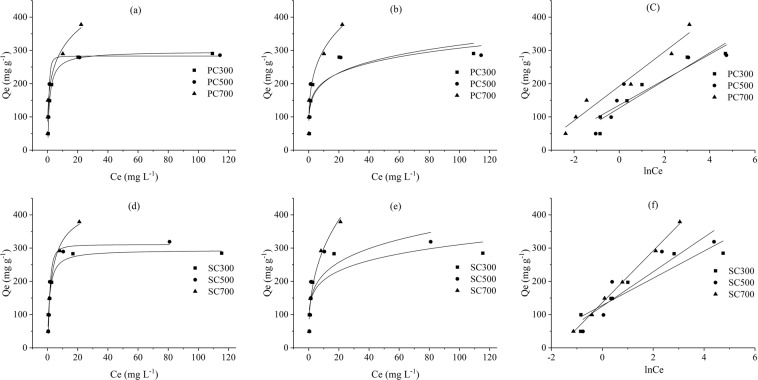
Langmuir (**a**), Freundlich (**b**) and Temkin(**c**) models for the PC samples. Langmuir (**d**), Freundlich (**e**) and Temkin (**f**) models for the SC samples.

In this study, the adsorption capacity of the biochars increased rapidly with increasing solution concentration in the low concentration range (0–10 mg L^−1^); when the solution concentration was further increased, the adsorption tended to reach equilibrium (Fig. 6). By comparing the linear correlation coefficients (R^2^) of the two models (Table 4), it was found that the adsorption behavior of the biochars can be better described by the Langmuir model, suggesting that the adsorption process tended to be monolayer adsorption^41^.

**Table 4:** Langmuir and Freundlich parameters for Pb(II) adsorption onto biochars.

Biochar	Langmuir			Freundlich			Temkin		
	Q_m_ (mg g^−1^)	K_L_ (L mg^−1^)	R^2^	K_F_ (mg g^−1^)(mg L^−1^)^1/n^	n	R^2^	A (L g^−1^)	b_T_ (J mol^−1^)	R^2^
PC300	294.86	0.760	0.963	133.75	5.47	0.717	21.04	117.78	0.881
PC500	293.67	1.015	0.927	137.58	5.74	0.692	34.64	71.52	0.790
PC700	326.74	2.315	0.947	165.90	3.83	0.938	40.99	60.44	0.945
SC300	294.86	0.760	0.963	133.75	5.47	0.717	23.04	60.02	0.844
SC500	332.63	0.582	0.926	135.28	4.66	0.700	11.18	47.22	0.823
SC700	398.06	0.470	0.982	130.57	2.77	0.956	5.74	31.41	0.977

The separation parameter *R*_*L*_ calculated based on ${R}_{L}=1/(1+{K}_{L}/{C}_{0})$ is usually used to describe the essential feature of the Langmuir model, where *K*_*L*_ is the Langmuir constant and *C*_0_ is the initial concentration of Pb(II) (mg L^−1^). When *R*_*L*_ = 0, adsorption is irreversible; when 0 < *R*_*L*_ < 1, adsorption is favorable; when *R*_*L*_ = 1, adsorption is linear; and when *R*_*L*_ > 1, adsorption is unfavorable. The *R*_*L*_ values of the SMS-derived biochars were in the range of 0.001–0.04 (between 0 and 1), implying that Pb(II) adsorption onto the SMS-derived biochars is favorable^42^. Studies have shown that a Freundlich constant, n, between 1 and 10 indicates favorable adsorption, and that the interactions between biochar and heavy metal ions are stronger when the n value is higher. In this study, the Freundlich constant n values ranged from 2.27 to 5.74, suggesting favorable adsorption.

According to the Langmuir model, the maximum adsorption capacities for the PC and SC samples were 326 and 398 mg g^−1^, respectively. The slightly higher adsorption capacities of the SC samples agree with the characterization analysis. The maximum adsorption capacities of Pb(II) onto the PC and SC samples were compared with those of reported biochars to assess their adsorption performance. The adsorption capacities of the biochars in this study were higher than those of the modified biochars in previous studies (Table 5).

**Table 5:** Maximum adsorption capacities of modified biochar in previous studies.

Feedstocks	Maximum adsorption capacities (mg g^−1^)	References
Rape straw	81.1	^25^
Sewage sludge	36.5	^27^
Pomelo peels	57.64	^28^
Rice straw	41.9	^29^
Tea branches	237.5	^30^
Spent *P. ostreatus* substrate	326	This study
Spent *shiitake* substrate	398	This study

### Mechanism of Pb(II) adsorption onto the biochars

**Figure 7:**
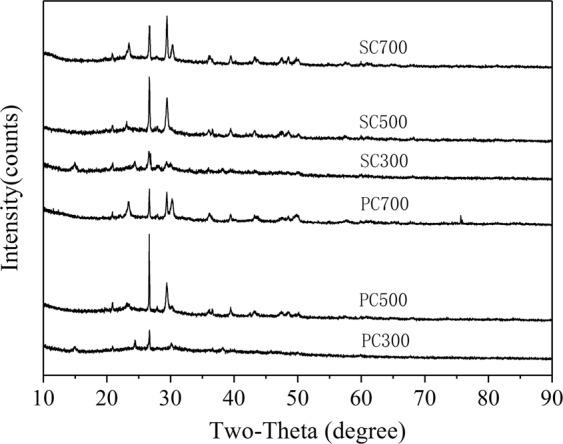
The XRD patterns of the biochars.

The alkalinity of the biochars induced by inorganic minerals could promote Pb(II) removal by forming a Pb(II) precipitate. The XRD patterns of the six biochars are shown in Fig. 7. The main peaks with the highest intensities are at 2θ = 26.73° and 2θ = 20.86°, confirming the presence of quartz. The peaks at 2θ = 29.43° and 2θ = 30.35° indicated the presence of CaCO_3_ and Ca_3_(PO_4_)_2_, respectively. A small peak at approximately 2θ = 15.00° for PC300 and SC300 could be ascribed to some crystalline cellulosic structures that did not decompose at low temperature^43^. The XRD analysis showed that the SMS-derived biochars contain CaCO_3_, Ca_3_(PO_4_)_2_ and several other substances; the inorganic anions (CO_3_^2−^, SO_4_^2−^ and OH^−^) might be released from the dissolved biochars, and they can take part in the precipitation process of Pb(II). According to other Pb(II) adsorption studies, the precipitates from the reaction of Pb^2+^ and inorganic anions can be observed in the XRD analysis after adsorption^44,45^. Therefore, the surface mineral precipitation of Pb(II) is a possible mechanism for Pb(II) adsorption onto the biochars.

**Figure 8:**
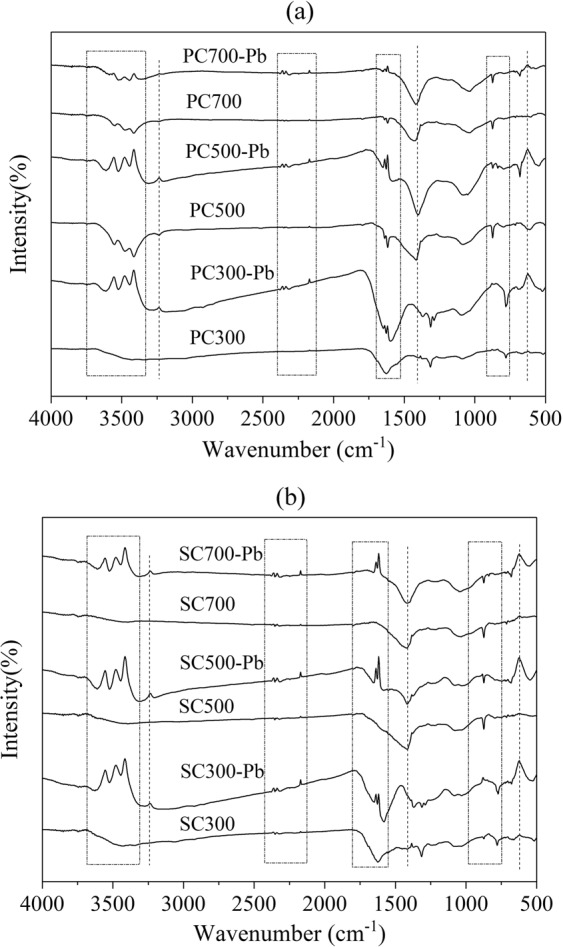
FTIR spectra of the PC and SC samples before and after their reaction with Pb(II).

The functional groups on a biochar affect the physicochemical properties and adsorption capacity of the biochar^46^. From the FTIR spectra of the Pb-free biochar in Fig. 8, it was found that the PC and SC samples have similar aromatic and aliphatic functional groups. The bands at 779~874 cm^−1^ were assigned to aromatic C-H groups preserved at high temperature (500 and 700 °C). The peaks at 1000–1100 cm^−1^ were associated with the C-O/C-O-C stretching vibrations of alcohols, phenols, and ether or ester groups; these oxygen-containing functional groups mainly originated from cellulose. The peak at 1632 cm^−1^ indicated the C=C ring stretching vibration of lignin connected to aromatic rings. The peak at 1688 cm^−1^ corresponded to the C=O groups. These aromatic structures could provide π-electrons, which may contribute to Pb(II) adsorption. The bands at ~3500 cm^−1^ represented the stretching vibrations of the O-H and N-H functional groups^47,48^. For the PC and SC samples, the intensity of the adsorption at 1616 cm^−1^ decreased at high pyrolysis temperatures, indicating the reduction of C=C and C=O in the aromatic rings, possibly due to the decomposition of lignin^49^.

The FTIR spectra of the biochars after adsorbing Pb(II) are shown in Fig. 8. It is obvious that the Pb(II) adsorption process changed the shapes and intensities of the bands in the FTIR spectra. The bands of the stretching O–H vibrations from 3200 cm^−1^ to 3600 cm^−1^ may be due to physisorbed water after adsorption^50^. A new peak for all the Pb-adsorbed biochars at ~650 cm^−1^ is indicative of Pb(II) adsorption onto biochar. For PC300 and SC300, the peak at 1626 cm^−1^ corresponding to the C=C and C=O bonds shifted to 1580 cm^−1^ after adsorption. These results indicated that the C=C and C=O rings may take part in the adsorption process, which can imply that the surface complexation and metal-π interactions were possible mechanisms^28^. Moreover, the peak of CO_3_^2−^ at ~1430 cm^−1^ for the Pb-adsorbed biochars convincingly demonstrated the formation of a precipitate between Pb^2+^ and CO_3_^2−^.

## Conclusions

SMSs can be ideal raw materials for the preparation of biochars. SMS-derived biochars became more aromatic with increasing pyrolysis temperature. The large surface area and numerous microporous structures of the biochars were beneficial for Pb(II) adsorption. The adsorption of Pb(II) is a chemisorption and monolayer adsorption process. The possible mechanisms may include the complexation between Pb(II) and the oxygenated functional groups, the formation of Pb^2+^-π interactions and the surface mineral precipitation of Pb(II). The biochars obtained at 700 °C have maximum adsorption capacities of 326 mg g^−1^ (PC) and 398 mg g^−1^ (SC), which are larger than those of many reported modified biochars. Overall, SMS-derived biochar is a promising and effective adsorbent for the removal of Pb(II) from water. Furthermore, this study provides a theoretical basis for the utilization of agricultural waste resources.
